# Supplementary material for: Genetic Diversity and Distribution of Italian Cave Crickets (Dolichopoda): Toward a Better Understanding of Lineage Structure
Source: Animals (Basel). 2025 Aug 19;15(16):2429. doi: 10.3390/ani15162429 (PMC12382646; doi:10.3390/ani15162429)
Supplement: Supplementary file 1 [file animals-15-02429-s001.zip › animals-3790586-supplementary.pdf]

Table S1. This table shows all the accession numbers of GenBank (GB) sequences within the genus *Dolichopoda* used in our phylogenetic analyses. The nomenclature of the species is retrieved from GB.

Accession Number (16S)	Accession Number (COI)	Species	Locality voucher	Cave	Locality	Country
AY793570	AY793600	*D. aegilion*	CAM	Miniera di Campese	Isola del Giglio, Grosseto, Toscana, Italy	IT
AY507572	AY507652	*D. aegilion*	CAM	Miniera di Campese	Isola del Giglio, Grosseto, Toscana, Italy	IT
EU887860a	EU887891	*D. annae*	OLI	Small caves	Kato Olimpos, Leptokaria, Kalipefki, Greece	GR
EU887860b	EU887892	*D. annae*	OLI	Small caves	Kato Olimpos, Leptokaria, Kalipefki, Greece	GR
EU887860c	EU887893	*D. annae*	OLI	Small caves	Kato Olimpos, Leptokaria, Kalipefki, Greece	GR
EU887861a	EU887894	*D. annae*	TEM	Aghlia Paraskevi Cave	Tembi Valley, Larissa, Greece	GR
EU887861b	EU887895	*D. annae*	TEM	Aghlia Paraskevi Cave	Tembi Valley, Larissa, Greece	GR
EU887861c	EU887896	*D. annae*	TEM	Aghlia Paraskevi Cave	Tembi Valley, Larissa, Greece	GR
	ON953153	*D. apollinea*	CND	Conoide cave	San Lorenzo Bellizzi, Cosenza, Calabria, Italy	IT
	ON953155	*D. apollinea*	FRA	Caprio cave	Francavilla marittima, Cosenza, Calabria, Italy	IT
	ON953156	*D. apollinea*	SAN	S. Angelo caves	Cassano allo Ionio, Cosenza, Calabria, Italy	IT
	ON953154	*D. apollinea*	SGU	Serra del Gufo	Cerchiara calabra, Cosenza, Calabria, Italy	IT
EF216974b	EF217018	*D. araneiformis*	SOL	Kod Solina Cave	Govedari, Miljet, Croatia	HR
	EU887889	*D. araneiformis*	SOL	Kod Solina Cave	Govedari, Miljet, Croatia	HR
EF216974a	EF217019	*D. araneiformis*	VEL	Velika Cave	Blato, Miljet, Croatia	HR
	EU887890	*D. araneiformis*	VEL	Velika Cave	Blato, Miljet, Croatia	HR
KM086477	KM086527	*D. azami*	BAL	Balmarossa Cave	Pradleves, Grana Valley, Cuneo, Piedmont	IT
KM086496	KM086546	*D. azami*	BAN	Bandito Cave	Valdieri, Gesso Valley, Cuneo, Piedmont, Italy	IT
KM086485	KM086535	*D. azami*	CHA	Mine close to Chauves-souris Cave	Chateaudouble, Var, France, type locality	FR
AY793571a	AY793639	*D. baccettii*	PST	Punta degli Stretti Cave	Orbetello, Grosseto, Toscana, Italy	IT
AY793571b	AY793640	*D. baccettii*	PST	Punta degli Stretti Cave	Orbetello, Grosseto, Toscana, Italy	IT
AY507593	AY507651	*D. baccettii*	PST	Punta degli Stretti Cave	Orbetello, Grosseto, Toscana, Italy	IT
AY507594	AY507650	*D. baccettii*	PST	Punta degli Stretti Cave	Orbetello, Grosseto, Toscana, Italy	IT
AY507579	AY507648	*D. bolivari*	FRN	Forat negre cueva	Serradell, Lleida, Pyrenees, Spain	ES
AY507580	AY507649	*D. bolivari*	FRN	Forat negre cueva	Serradell, Lleida, Pyrenees, Spain	ES

AY793578a	AY793631	*D. bormansi*	BRA	Brando Cave	Bastia, Corsica, France	FR
AY793578b	AY793632	*D. bormansi*	BRA	Brando Cave	Bastia, Corsica, France	FR
AY793578c	AY793627	*D. bormansi*	BRA	Brando Cave	Bastia, Corsica, France	FR
AY507605	AY507646	*D. bormansi*	BRA	Brando Cave	Bastia, Corsica, France	FR
AY507606	AY507647	*D. bormansi*	BRA	Brando Cave	Bastia, Corsica, France	FR
AY793579a	AY793625	*D. bormansi*	SIS	Sisco Cave	Corsica Island, France	FR
AY793579b	AY793626	*D. bormansi*	SIS	Sisco Cave	Corsica Island, France	FR
AY793579c	AY793628	*D. bormansi*	SIS	Sisco Cave	Corsica Island, France	FR
	ON953160	*D. calabra*	MIL	Milogno cave	Piemontese, Cosenza, Calabria, Italy	IT
	ON953157	*D. calabra*	MON	Monaca cave	S. Agata di Esaro, Cosenza, Calabria, Italy	IT
	ON953159	*D. calabra*	RIZ	Rizzo cave	Cetraro, Cosenza, Calabria, Italy	IT
	ON953158	*D. calabra*	TES	Tesauro cave	S. Agata di Esaro, Cosenza, Calabria, Italy	IT
EF216963	EF217047	*D. calidnae*	EPT	Seven Virgins Cave	Kalimnos, Kalimnos Island, Dodecanese, Greece	GR
EF216964	EF217048	*D. calidnae*	SKA	Skalia cave	Skalia, Kalimnos Island, Dodecanese, Greece	GR
AY793587a	AY793606	*D. capreensis*	CPR	San Michele Cave	Isola di Capri, Napoli, Campania, Italy	IT
AY793587b	AY793607	*D. capreensis*	CPR	San Michele Cave	Isola di Capri, Napoli, Campania, Italy	IT
AY507574	AY507644	*D. capreensis*	CPR	San Michele Cave	Isola di Capri, Napoli, Campania, Italy	IT
EF216961a	EF217035	*D. cassagnaui*	TRI	Aghlia Triada Cave	Karistos, Eubea Island, Greece	GR
EF216961b	EF217036	*D. cassagnaui*	TRI	Aghlia Triada Cave	Karistos, Eubea Island, Greece	GR
EF216961c	EF217037	*D. cassagnaui*	TRI	Aghlia Triada Cave	Karistos, Eubea Island, Greece	GR
AY793576a	AY793618	*D. cyrnensis*	SAB	Sabara Cave	Corsica Island, France	FR
AY793576b	AY793619	*D. cyrnensis*	SAB	Sabara Cave	Corsica Island, France	FR
AY793577a	AY793620	*D. cyrnensis*	VLT	Valletto Cave	Corsica Island, France	FR
AY793577b	AY793621	*D. cyrnensis*	VLT	Valletto Cave	Corsica Island, France	FR
MW358532	MW357334	*D. dalensi*	DER	Dervenaki cave	Nemea, Korinthia, Greece	GR
MW358534	MW357336	*D. dalensi*	KAD	Kaliakoudotripa	Nemea, Korinthia, Greece	GR
EF216959	EF217026	*D. dalensi*	KEF	Kefalovrisi Cave	Argos, Argolide, Greece	GR
MW358533	MW357335	*D. dalensi*	NES	Nestani	Katavothra Nestani, Arcadia, Greece	GR
MW358530	MW357332	*D. epidavrii*	MOK	Spilaio Moni Kalamiou	Epidauros, Argolida, Greece	GR

MW358529	MW357331	*D. epidavrii*	MOK	Spilaio Moni Kalamiou	Epidauros, Argolida, Greece	GR
MW358528	MW357330	*D. epidavrii*	MOK	Spilaio Moni Kalamiou	Epidauros, Argolida, Greece	GR
MW358527	MW357329	*D. epidavrii*	MOK	Spilaio Moni Kalamiou	Epidauros, Argolida, Greece	GR
AY793565	AY793623	*D. euxina*	GOL	Golova Otapa Cave	Caucasus, Russia	RU
AY793566	AY793622	*D. euxina*	VOR	Vorontzovskaya Cave	Caucasus, Russia	RU
EF216950a	EF217008	*D. gasparoi*	CHI	Chirospilia Cave	Evghiros, Levkada, Greece	GR
EF216950b	EF217009	*D. gasparoi*	CHI	Chirospilia Cave	Evghiros, Levkada, Greece	GR
AY507566	AY507611	*D. geniculata*	AUS	Grotta degli ausi	Prossedi, Latina, Lazio, Italy	IT
AY507563	AY507609	*D. geniculata*	CLP	Grotta Regina Margherita	Collepardo, Frosinone, Lazio, Italy	IT
AY793584	AY793594	*D. geniculata*	FON	Fontanelle Cave	Campania, Italy	IT
AY793585	AY793595	*D. geniculata*	ISC	Ischia cellars	Isola di Ischia, Napoli, Campania, Italy	IT
AY507581	AY507622	*D. geniculata*	ISC	Fontana cunicoli	Isola di Ischia, Napoli, Campania, Italy	IT
AY507586	AY507621	*D. geniculata*	PAS	Grotta di Pastena	Pastena, Frosinone, Lazio, Italy	IT
AY507587	AY507620	*D. geniculata*	PIL	Grotta la Pila	Poggio Moiano, Rieti, Lazio, Italy	IT
AY507590	AY507618	*D. geniculata*	PRA	Grotta delle Praie	Lettomanoppello, Perugia, Umbria, Italy	IT
AY507599	AY507624	*D. geniculata*	TUS	Cunicolo dell'acquedotto	Frascati, Roma, Lazio, Italy	IT
AY507600	AY507625	*D. geniculata*	VAL	Valmarino Cave	Monte S. Biagio, Latina, Lazio, Italy	IT
AY793583a	AY793616	*D. geniculata*	VAL	Valmarino Cave	Monte S. Biagio, Latina, Lazio, Italy	IT
AY793583b	AY793617	*D. geniculata*	VAL	Valmarino Cave	Monte S. Biagio, Latina, Lazio, Italy	IT
KY426928	KY426937	*D. geniculata*	VAL	Valmarino Cave	Monte S. Biagio, Latina, Lazio, Italy	IT
Y07551		*D. geniculata*	Y07551			
AY793586a	AY793596	*D. geniculata pontiana*	PNZ	Roman Aqueduct	Isola di Ponza, Latina, Lazio, Italy	IT
AY793586b	AY793597	*D. geniculata pontiana*	PNZ	Roman Aqueduct	Isola di Ponza, Latina, Lazio, Italy	IT
AY507588	AY507619	*D. geniculata pontiana*	PNZ	Le Forme	Isola di Ponza, Latina, Lazio, Italy	IT
EF216952	EF217012	*D. giachinoi*	ORO	Megalospilio Cave	Monastirakion, Aitolo-Akarnania, Greece	GR
	EU887897	*D. giachinoi*	ORO	Megalospilio Cave	Monastirakion, Aitolo-Akarnania, Greece	GR
EF216965	EF217049	*D. giulianae*	SPS	Moni Spilianis Cave	Pithagorion, Samos Island, Greece	GR
EF216953	EF217013	*D. graeca*	PER	Perama Cave	Ioannina, Epiro, Greece	GR
AY507607	AY507615	*D. hussoni*	HEL	Naoussa cave	Naoussa, Macedonia, Greece	GR
EF216973a	EF217031	*D. hussoni*	IZB	Apano Skala Cave	Naoussa, Imathia, Greece	GR

EF216973b	EF217032	*D. hussoni*	NAU	Saranda Outdate Cave	Naoussa, Imathia, Greece	GR
EF216973c	EF217033	*D. hussoni*	NAU	Saranda Outdate Cave	Naoussa, Imathia, Greece	GR
EF216973d	EF217034	*D. hussoni*	NAU	Saranda Outdate Cave	Naoussa, Imathia, Greece	GR
EU887871	EU887919	*D. hyrcana*	LEN	Azerbaijan	Lenkoran, East Caucaso, Türkiye	TR
EF216968	EF217054	*D. insignis*	PAN	Panos Cave	Marathon, Athene, Attica, Greece	GR
EF216949a	EF217006	*D. ithakii*	ITA	Marmarospilia cave	Vathi, Ithaki Island, Greece	GR
EF216949b	EF217007	*D. ithakii*	ITA	Marmarospilia cave	Vathi, Ithaki Island, Greece	GR
EF216954a	EF217014	*D. kiriakii*	AGH	Kiriaki Cave	Korifè, Aghlia Kiriaki, Parga, Greece	GR
EF216954b	EF217015	*D. kiriakii*	AGH	Kiriaki Cave	Korifè, Aghlia Kiriaki, Parga, Greece	GR
MW358540	MW357342	*D. kofinasi*	DIN	Leonidio	Spilia Dionysou, Arcadia, Greece	GR
MW358536	MW357338	*D. kofinasi*	KAP	Spilaio Kapsia	Kapsia, Arcadia, Greece	GR
MW358541	MW357343	*D. kofinasi*	KOS	Kosmas	Spilaio Kosma, Arcadia , Greece	GR
MW358543	MW357345	*D. kofinasi*	PIG	Spilaio Pigaza	Vellies, Laconia, Greece	GR
MW358542	MW357344	*D. kofinasi*	SFE	Barathro Sfendami	Monemvassia, Laconia, Greece	GR
MW358535	MW357337	*D. kofinasi*	TOU	Katavothra Tousi	Kapsia, Arcadia, Greece	GR
MW358539	MW357341	*D. kofinasi*	TRK	Spilaio Trupitses	Skortsinos, Arcadia, Greece	GR
MW358538	MW357340	*D. kofinasi*	TTS	Spilaio Tyrias	Kollines, Arcadia, Greece	GR
MW358537	MW357339	*D. kofinasi*	TTS	Spilaio Tyrias	Kollines, Arcadia, Greece	GR
MW358544	MW357346	*D. kofinasi*	VRI	Spilaio Vri	Monemvassia, Laconia, Greece	GR
	AY507608	*D. laetitiae*	FOR	Ruderi di Villa Chigi	Formello, Roma, Lazio, Italy	IT
AY793582a	AY793612	*D. laetitiae*	GDP	Piane Cave	Umbria, Italy	IT
AY793582b	AY793610	*D. laetitiae*	GDP	Piane Cave	Umbria, Italy	IT
AY507591	AY507641	*D. laetitiae*	PSC	Poscola Cave	Monte di Malo, Priabona, Vicenza, Veneto, Italy	IT
AY507592	AY507642	*D. laetitiae*	PSC	Poscola Cave	Monte di Malo, Priabona, Vicenza, Veneto, Italy	IT
AY793581a	AY793611	*D. laetitiae*	PSC	Poscola Cave	Monte di Malo, Priabona, Vicenza, Veneto, Italy	IT
AY793581b	AY793613	*D. laetitiae*	PSC	Poscola Cave	Monte di Malo, Priabona, Vicenza, Veneto, Italy	IT
KY426929	KY426938	*D. laetitiae*	PSC	Poscola Cave	Monte di Malo, Priabona, Vicenza, Veneto, Italy	IT
AY793580a	AY793614	*D. laetitiae etrusca*	DIA	Diavolo Cave	Semproniano, Grosseto, Toscana, Italy	IT

AY793580b	AY793615	*D. laetitiae etrusca*	DIA	Diavolo Cave	Semproniano, Grosseto, Toscana, Italy	IT
AY507578	AY507640	*D. laetitiae etrusca*	DIA	Diavolo Cave	Semproniano, Grosseto, Toscana, Italy	IT
KM086489	KM086539	*D. ligustica*	ART	Taragnina Cave	Balestrino, Savona, Liguria, Italy, close to Santa Lucia Inferiore Cave	IT
KM086484	KM086534	*D. ligustica*	BES	Besta Cave	Vievola, Tenda, France	FR
KM086491	KM086541	*D. ligustica*	BLO	Buco delle Locuste Cave	Pagliano, Varaita Valley, Cuneo, Piedmont, Italy	IT
KM086478	KM086528	*D. ligustica*	BMA	Buco del Maestro Cave	Paesana, Po Valley, Cuneo, Piedmont, Italy	IT
AY507569	AY507612	*D. ligustica*	BOS	Bossea Cave	Frabosa Soprana, Corsaglia Valley, Cuneo, Piedmont, Italy	IT
AY507570	AY507613	*D. ligustica*	BOS	Bossea Cave	Frabosa Soprana, Corsaglia Valley, Cuneo, Piedmont, Italy	IT
KM086490	KM086540	*D. ligustica*	BOS	Bossea Cave	Frabosa Soprana, Corsaglia Valley, Cuneo, Piedmont, Italy	IT
KM086480	KM086530	*D. ligustica*	BPR	Buco del Partigiano Cave	Roccabruna, Maira Valley, Cuneo, Piedmont, Italy	IT
AY793568a	AY793604	*D. ligustica*	CON	Corno Cave	Piemonte, Western-North Italy	IT
AY793568b	AY793605	*D. ligustica*	CON	Corno Cave	Piemonte, Western-North Italy	IT
AY507576	AY507614	*D. ligustica*	CORN	Buco del Corno	Valle Cavallina, Zandobbio, Bergamo lombardia, Italy	IT
KM086497	KM086547	*D. ligustica*	GOR	Orso Cave	Ponte di Nava, Ormea, Cuneo, Piedmont, Italy	IT
KM086494	KM086544	*D. ligustica*	GPR	Partigiani Cave	Rossana, Varaita Valley, Cuneo, Piedmont, Italy	IT
KM086495	KM086545	*D. ligustica*	LIG	Viozene	Ormea, upper Tanaro Valley, Cuneo, Piedmont, Italy	IT
KM086482	KM086532	*D. ligustica*	PGA	Gaiola Cave	Gaiola, Stura di Demonte Valley, Cuneo, Piedmont, Italy	IT
KM086492	KM086542	*D. ligustica*	RIT	Rittana Cave	Rittana, Stura di Demonte Valley, Cuneo, Piedmont, Italy	IT
KM086488	KM086538	*D. ligustica*	SBA	Bandito hypogeum	Roaschia, Gesso Valley, Cuneo, Piedmont, Italy	IT
AY507597	AY507623	*D. ligustica*	SFL	Grotta Selva	Zandobbio, Bergamo, Lombardia, Italy	IT
KM086483	KM086533	*D. ligustica*	SVE	Vernante hypogeum	Vernante, Vermenagna Valley, Cuneo, Piedmont, Italy	IT

KM086487	KM086537	*D. ligustica*	SVS	Vallone Saben eastern hypogeum	Valdieri, Gesso Valley, Cuneo, Piedmont, Italy	IT
KM086493	KM086543	*D. ligustica*	TDR	Dronera Cave	Vicoforte Mondovì, Ermetta Valley, Cuneo, Piedmont, Italy	IT
KM086479	KM086529	*D. ligustica septentrionalis*	BCS	Borna del Servais Cave	Ala di Stura, Ala Valley, Torino, Piedmont, Italy	IT
KM086481	KM086531	*D. ligustica septentrionalis*	CHI	Chiabrano Cave	Perrano, Germanasca Valley, Torino, Piedmont, Italy	IT
KM086498	KM086548	*D. ligustica septentrionalis*	CVF	Villar Focchiardo Cave	Villar Focchiardo, Susa Valley, Torino, Piedmont, Italy	IT
KM086486	KM086536	*D. ligustica septentrionalis*	FAR	Farout Cave	Pradleves, Grana Valley, Cuneo, Piedmont, Italy	IT
AY793569a	AY793602	*D. ligustica septentrionalis*	PUG	Borna Maggiore del Pugnetto Cave	Mezzenile, Lanzo Valley, Torino, Piedmont, Italy, type locality	IT
AY793569b	AY793601	*D. ligustica septentrionalis*	PUG	Borna Maggiore del Pugnetto Cave	Mezzenile, Lanzo Valley, Torino, Piedmont, Italy, type locality	IT
AY793569c	AY793603	*D. ligustica septentrionalis*	PUG	Borna Maggiore del Pugnetto Cave	Mezzenile, Lanzo Valley, Torino, Piedmont, Italy, type locality	IT
KM086499	KM086549	*D. ligustica septentrionalis*	PUG	Borna Maggiore del Pugnetto Cave	Mezzenile, Lanzo Valley, Torino, Piedmont, Italy, type locality	IT
AY507595	AY507616	*D. ligustica septentrionalis*	PUG	Borna Maggiore del Pugnetto Cave	Mezzenile, Lanzo Valley, Torino, Piedmont, Italy, type locality	IT
AY507596	AY507617	*D. ligustica septentrionalis*	PUG	Borna Maggiore del Pugnetto Cave	Mezzenile, Lanzo Valley, Torino, Piedmont, Italy, type locality	IT
KM086500	KM086550	*D. ligustica septentrionalis*	VDT	Val della Torre Mine	Val della Torre, Casternone Valley, Torino, Piedmont, Italy	IT
KM086501	KM086551	*D. ligustica septentrionalis*	ZAN	Lacca Selva Cave	Zandobbio, Bergamo, Lombardy, Italy	IT
KY426927	KY426936	*D. linderi*	BNP	Grotte de Bon Repaux	Bon Repaux, Ariege,Pyrenees, France	FR
AY507565	AY507626	*D. linderi*	BNP	Grotte de Bon Repaux	Bon Repaux, Ariege,Pyrenees, France	FR
AY507583	AY507627	*D. linderi*	MTB	Grotte de Montbolo	Montbolo, Eastern Pyrenees, France	FR
AY793567a	AY793598	*D. linderi*	SIR	Sirach Cave	Eastern Pyrenees, Western-South France	FR
AY793567b	AY793599	*D. linderi*	SIR	Sirach Cave	Eastern Pyrenees, Western-South France	FR
AY507603	AY507628	*D. linderi*	VMY	Grotte de Valmanya	Vinca, Eastern Pyrenees, France	FR

EU887863	EU887901	*D. lustriae*	AND	Aghios Andreas Cave	Valtou M., Halkiopuli, Etolia, Greece	GR
	JX952180	*D. lycia*	601BurS10	Antalya Kemer	Türkiye	TR
KC783270		*D. lycia*	GEY	Geyikbayırı Cave	Geyikbayırı, Antalya, Türkiye	TR
KC783271		*D. lycia*	SIT	Sıtmaini Cave	Gedelma, Kemer, Antalya, Türkiye	TR
EU887870	EU887918	*D. lycia*	TUR	Gedelma Cave	Gedelma village, Kermer, Antalya, Türkiye	TR
EF216971b	EF217041	*D. makrykapa*	KSA	Paralia Pot Cave	Kao Seta, Aghlia Triada, Eubea Island, Greece	GR
EF216971a	EF217042	*D. makrykapa*	PKI	Paralia Kilidau Cave	Lamari, Eubea Island, Greece	GR
EF216957a	EF217022	*D. matsakisi*	ANA	Analipsi Cave	Pititsa, Achaia, Greece	GR
EF216957b	EF217023	*D. matsakisi*	ANA	Analipsi Cave	Pititsa, Achaia, Greece	GR
EF216957c	EF217024	*D. matsakisi*	KAS	Ton Limnon Cave	Kastri, Kalavrita, Achaia, Greece	GR
MW358531	MW357333	*D. matsakisi*	KAS	Ton Limnon Cave	Kastri, Kalavrita, Achaia, Greece	GR
AY793575a	AY793629	*D. muceddai*	SAR	Monte Limbara	Sardinia, Italy	IT
AY793575b	AY793630	*D. muceddai*	SAR	Monte Limbara	Sardinia, Italy	IT
EU887867	EU887908	*D. naxia*	STA	small Cave	Stauros, Naxos Island, Cyklades, Greece	GR
EU887868a	EU887909	*D. naxia*	ZEU	Zeus Cave	Filotas, Naxos Island, Cyklades, Greece	GR
EU887868b	EU887910	*D. naxia*	ZEU	Zeus Cave	Filotas, Naxos Island, Cyklades, Greece	GR
EU887868c	EU887911	*D. naxia*	ZEU	Zeus Cave	Filotas, Naxos Island, Cyklades, Greece	GR
KC783278		*D. noctivaga*	ANA	Anaçbaşı Cave	Kabadüz, Ordu, Türkiye	TR
EU887872	EU887920	*D. noctivaga*	COR	Coruh Valley	Ispir, Erzurum district, Türkiye	TR
EU887873	EU887921	*D. noctivaga*	DUZ	Duzkoy	Artvin district, Türkiye	TR
EU887874	EU887922	*D. noctivaga*	KAFB	Kafkasor	Artvin district, Türkiye	TR
KC783277		*D. noctivaga*	KEL	Kelemen Cave	Kelemen, Yenice, Karabük, Türkiye	TR
KC783276		*D. noctivaga*	SAR	Sarıkaya Cave	Yığılca, Bolu, Türkiye	TR
KC783279		*D. noctivaga*	ZEF	Zefre Cave	Espiye, Gireseun, Türkiye	TR
MK993655		*D. octhoniai*	OCT	Graspilea Cave	Ochtonia, Eubea Island, Greece	GR
	ON953163	*D. palpata*	ACS	Sponza Aqueduct	Verzino, Crotone, Calabria, Italy	IT
	ON953162	*D. palpata*	GRU	Grave Grubbo	Verzino, Crotone, Calabria, Italy	IT
	ON953167	*D. palpata*	LUM	Lamia cave	Montbello Ionico, Reggio Calabria, Calabria, Italy	IT
	ON953164	*D. palpata*	MFR	Du Manfred cave	Verzino, Crotone, Calabria, Italy	IT

	ON953161	*D. palpata*	MLG	Mine	Longobucco, Cosenza, Calabria, Italy	IT
AY793588a	AY793608	*D. palpata*	TRE	Tremusa cave	Scilla, Reggio di Calabria, Calabria, Italy	IT
AY793588b	AY793609	*D. palpata*	TRE	Tremusa cave	Scilla, Reggio di Calabria, Calabria, Italy	IT
AY507564	AY507610	*D. palpata*	TRE	Tremusa cave	Scilla, Reggio di Calabria, Calabria, Italy	IT
AY507598	AY507645	*D. palpata*	TRE	Tremusa cave	Scilla, Reggio di Calabria, Calabria, Italy	IT
	ON953166	*D. palpata*	TRE	Tremusa cave	Scilla, Reggio di Calabria, Calabria, Italy	IT
	ON953165	*D. palpata*	TRE	Tremusa cave	Scilla, Reggio di Calabria, Calabria, Italy	IT
EF216972c	EF217030	*D. paraskevi*	NIK	Atzigano cave	Adrianos, Lassithi, Greece	GR
	EU887912	*D. paraskevi*	NIK	Atzigano cave	Adrianos, Lassithi, Greece	GR
EF216972a	EF217027	*D. paraskevi*	PAR	Aghlia Paraskevi Cave	Skotinon, Iraklio, Greece	GR
EF216972b	EF217028	*D. paraskevi*	PAR	Aghlia Paraskevi Cave	Skotinon, Iraklio, Greece	GR
EU887862a	EU887898	*D. patrizii*	PET	Small cave	Petalas, Greece	GR
EU887862b	EU887899	*D. patrizii*	PET	Small cave	Petalas, Greece	GR
EU887862c	EU887900	*D. patrizii*	PET	Small cave	Petalas, Greece	GR
EF216951a	EF217010	*D. pavesii*	SPI	Drogarati Cave	Sami, Kefalonia Island, Greece	GR
EF216951b	EF217011	*D. pavesii*	SPI	Drogarati Cave	Sami, Kefalonia Island, Greece	GR
	EF217053b	*D. petrochilosi*	DHA	Aghlia Joannis	Nea Pendeli, Athene, Attica, Greece	GR
EF216967a	EF217051	*D. petrochilosi*	JOA	Aghlia Joannis	Nea Pendeli, Athene, Attica, Greece	GR
EF216967b	EF217052	*D. petrochilosi*	JOA	Aghlia Joannis	Nea Pendeli, Athene, Attica, Greece	GR
EF216967c	EF217053a	*D. petrochilosi*	JOA	Aghlia Joannis	Nea Pendeli, Athene, Attica, Greece	GR
MW358554	MW357356	*D. poseidonica*	BAL	Balli Cave	Kardamili, Messinia	GR
MW358556	MW357358	*D. poseidonica*	LIC	Licurgo Cave	Kardamili, Messinia, Greece	GR
MW358555	MW357357	*D. poseidonica*	LIC	Licurgo Cave	Kardamili, Messinia, Greece	GR
MW358557	MW357359	*D. poseidonica*	MAN	Katafigi Mantagari	Kardamili, Messinia, Greece	GR
MW358553	MW357355	*D. propantii*	PRO	Spilaio Propanti	Andritsena, Ilia, Greece	GR
MW358552	MW357354	*D. propantii*	PRO	Spilaio Propanti	Andritsena, Ilia, Greece	GR
MW358551	MW357353	*D. propantii*	PRO	Spilaio Propanti	Andritsena, Ilia, Greece	GR
KC783275		*D. pusilla*	AKB	Akbez Cave	Akbez, Hatay, Türkiye	TR
AY793589	AY793637a	*D. remyi*	EDE	Waterfalls Cave	Edessa, Pella, Greece	GR
EF216969a	AY793637c	*D. remyi*	EDE	Waterfalls Cave	Edessa, Pella, Greece	GR

AY793590	AY793638	*D. remyi*	POZ	Pozarska Mala Pestera	Loutrakiou, Pella, Greece	GR
EF216969b	AY793637b	*D. remyi*	POZ	Pozarska Mala Pestera	Loutrakiou, Pella, Greece	GR
MK993656		*D. saraolacosi*	SARs	Abandoned mine	Atsitsa, Skyros island, Greece	GR
	JX952181	*D. sbordonii*	576AntS10	Burdur	Türkiye	TR
EU887869	EU887917	*D. sbordonii*	INS	Insuyu Cave	Burdur, Türkiye	TR
EF216966	EF217050	*D. sbordonii*	KAR	Karain Cave	Döşemealtı, Antalya, Türkiye	TR
KC783272		*D. sbordonii*	KAR	Musaini Cave	Döşemealtı, Antalya, Türkiye	TR
KC783274		*D. sbordonii*	KOC	Kocain Cave	Karaveliler, Türkiye	TR
KC783273		*D. sbordonii*	TAB	Tabak Cave	Kırkgözler, Antalya, Türkiye	TR
AY507567	AY507629	*D. schiavazzii*	BDO	Grotta di Buca dell’oro	Isola d’Elba, Grosseto, Toscana, Italy	IT
AY507568	AY507630	*D. schiavazzii*	BDO	Grotta di Buca dell’oro	Isola d’Elba, Grosseto, Toscana, Italy	IT
AY507571	AY507643	*D. schiavazzii*	BSC	Buca sopra cimitero	Orbetello, Grosseto, Toscana, Italy	IT
AY507573	AY507631	*D. schiavazzii*	CISC	Acquedotto di Cisternino	Livorno, Toscana, Italy	IT
AY507575	AY507632	*D. schiavazzii*	CPS	Monastero dei Fratelli Passionisti	Orbetello, Grosseto, Toscana, Italy	IT
AY793572	AY793635	*D. schiavazzii*	MRC	Marciana Cave	Isola d’Elba, Grosseto, Toscana, Italy	IT
AY507582	AY507634	*D. schiavazzii*	MRC	Marciana Cave	Isola d’Elba, Grosseto, Toscana, Italy	IT
AY793573	AY793633	*D. schiavazzii*	ORS	Pipistrelli Cave	Montorsaio, Grosseto, Toscana, Italy	IT
AY507584	AY507636	*D. schiavazzii*	ORS	Pipistrelli Cave	Montorsaio, Grosseto, Toscana, Italy	IT
AY507585	AY507635	*D. schiavazzii*	ORS	Pipistrelli Cave	Montorsaio, Grosseto, Toscana, Italy	IT
AY507589	AY507637	*D. schiavazzii*	POP	Populonia	Grosseto, Toscana, Italy	IT
AY507601	AY507638	*D. schiavazzii*	VET	Necropoli di Vetulonia	Grosseto, Toscana, Italy	IT
AY507602	AY507639	*D. schiavazzii*	VET	Necropoli di Vetulonia	Grosseto, Toscana, Italy	IT
AY793574a	AY793636	*D. schiavazzii caprai*	FIC	Fichino Cave	Cascianna Terme, Pistoia, Toscana, Italy	IT
AY793574b	AY793634	*D. schiavazzii caprai*	FIC	Fichino Cave	Cascianna Terme, Pistoia, Toscana, Italy	IT
AY507577	AY507633	*D. schiavazzii caprai*	FIC	Fichino Cave	Cascianna Terme, Pistoia, Toscana, Italy	IT
EF216960	EF217029	*D. sp. Crete*	DHI	Dikteion Antron Cave	Psychro, Iraklio, Greece	GR
	EU887913	*D. sp. East Aegean*	SPS	Moni Spilianis Cave	Pithagorion, Samos Island, Greece	GR
	EU887914	*D. sp. East Aegean*	SPS	Moni Spilianis Cave	Pithagorion, Samos Island, Greece	GR
	EU887916	*D. sp. East Aegean*	SPS	Moni Spilianis Cave	Pithagorion, Samos Island, Greece	GR

	EU887915	*D. sp. East Aegean*	SPS	Moni Spilianis Cave	Pithagorion, Samos Island, Greece	GR
EF216958	EF217025	*D. sp. Limnon*	KAS	Ton Limnon Cave	Kastri, Kalavrita, Achaia, Greece	GR
	EU887907	*D. sp. Limnon*	KAS	Ton Limnon Cave	Kastri, Kalavrita, Achaia, Greece	GR
EU887866a	EU887905	*D. sp. Parnaso*	KOR	Korykion Andron Cave	Mount Parnitha, Attica, Greece	GR
EU887866b	EU887906	*D. sp. Parnaso*	KOR	Korykion Andron Cave	Mount Parnitha, Attica, Greece	GR
EU887864a	EU887902	*D. sp. Taygeto*	TAY	Taygeto Shelter	Parori, Sparta, Lakonia, Greece	GR
EU887864b	EU887903	*D. sp. Taygeto*	TAY	Taygeto Shelter	Parori, Sparta, Lakonia, Greece	GR
EU887865	EU887904	*D. sp. Taygeto*	VAR	Aghia Varvara Cave	Parori, Sparta, Lakonia (Taygeto Mt), Greece	GR
EF216955a	EF217016	*D. steriotisi*	ANT	Antropograva Cave	Klimatia, Kerkira, Corfu, Greece	GR
EF216955b	EF217017	*D. steriotisi*	ANT	Antropograva Cave	Klimatia, Kerkira, Corfu, Greece	GR
KC783269		*D. sutini*	ASK	Aşıkali Cave	Söke, Aydın, Türkiye	TR
KC783268		*D. sutini*	SUT	Sütini Cave	Selçuk, İzmir, Türkiye	TR
EF216956a	EF217020	*D. thasosensis*	DRA	Drakotripa Cave	Panayia, Thasos Island, Kavala, Greece	GR
EF216956b	EF217021	*D. thasosensis*	DRA	Drakotripa Cave	Panayia, Thasos Island, Kavala, Greece	GR
MW358546	MW357348	*D. unicolor*	ALE	Alepotripa Cave	Dirou, Aeropolis, Laconia, Greece	GR
EF216970a	EF217043	*D. unicolor*	DIR	River cave of Glyfada	Dirou, Aeropolis, Laconia, Greece	GR
EF216970b	EF217044	*D. unicolor*	DIR	River cave of Glyfada	Dirou, Aeropolis, Laconia, Greece	GR
MW358547	MW357349	*D. unicolor*	GRO	Cave 2 Kastorio	Taygetos, Laconia, Greece	GR
EF216970c	EF217045	*D. unicolor*	KAT	Kataphingi Cave	Selitsa, Messenia, Greece	GR
EF216970d	EF217046	*D. unicolor*	KAT	Kataphingi Cave	Selitsa, Messenia, Greece	GR
MW358548	MW357350	*D. unicolor*	KOU	Koukouri cave	Kafionas, Laconia, Greece	GR
MW358545	MW357347	*D. unicolor*	SEL	Katafygi Cave	Selinitsa, Messinia, Greece	GR
MW358549	MW357351	*D. unicolor*	SKR	Skreti cave	Kastorio, Taygetos, Laconia, Greece	GR
MW358550	MW357352	*D. unicolor*	VRD	Vordionatiki cave	Kastorio, Taygetos, Laconia, Greece	GR
EF216962c	EF217038	*D. vandeli*	GLK	Cave over Kopais Lake	Orkomenos, Beotia, Greece	GR
EF216962a	EF217039	*D. vandeli*	HER	Hermes Cave	Orkomenos, Dhionisos, Beotia, Greece	GR
EF216962b	EF217040	*D. vandeli*	HER	Hermes Cave	Orkomenos, Dhionisos, Beotia, Greece	GR
